# Increased Effusion Synovitis for Those With a Dysregulated Inflammatory Response After an Anterior Cruciate Ligament Injury

**DOI:** 10.7759/cureus.37862

**Published:** 2023-04-20

**Authors:** Cale A Jacobs, Austin V Stone, Caitlin E. W Conley, Varag Abed, Janet L Huebner, Virginia B Kraus, Stacy E Smith, Christian Lattermann

**Affiliations:** 1 Orthopaedic Surgery, Brigham & Women’s Hospital, Boston, USA; 2 Orthopaedic Surgery and Sports Medicine, University of Kentucky, Lexington, USA; 3 Molecular Physiology Institute, Duke University School of Medicine, Durham, USA; 4 Radiology, Brigham & Women’s Hospital, Boston, USA

**Keywords:** magnetic resonance imaging, biomarker, cartilage, ligament, inflammation, knee

## Abstract

Introduction

The progression to posttraumatic osteoarthritis (PTOA) after an anterior cruciate ligament (ACL) injury is likely multifactorial, involving biological, mechanical, and psychosocial factors. Following acute joint trauma, there appears to be a subset of patients that demonstrate a dysregulated inflammatory response. This pro-inflammatory phenotype, or “Inflamma-type,” is characterized by an amplified pro-inflammatory response combined with a lack of attendant anti-inflammatory response and has been observed following both an ACL injury and an intra-articular fracture. The aims of this study were to: 1) compare magnetic resonance imaging (MRI)-measured effusion synovitis between those with vs. without a dysregulated inflammatory response, and 2) assess the correlations between effusion synovitis and synovial fluid concentrations of proinflammatory cytokines, degradative enzymes, and synovial fluid biomarkers of cartilage degradation.

Methods

A cluster analysis was previously performed with synovial fluid concentrations of biomarkers of inflammation and cartilage degradation from 35 patients with acute ACL injuries. Patients were then categorized into two groups: a pro-inflammatory phenotype (“Inflamma-type”) and those with a more normal inflammatory response to injury (NORM). Effusion synovitis measured from each patient’s preoperative clinical MRI scan was compared between the Inflamma-type and NORM groups using an independent, two-tailed t-test. In addition, Spearman’s rho non-parametric correlations were calculated to evaluate the relationship between effusion synovitis and each of the synovial fluid concentrations of pro-inflammatory cytokines, degradative enzymes, and biomarkers of cartilage degradation and bony remodeling.

Results

Effusion synovitis was significantly greater for the Inflamma-type (10.9±3.8 mm) than the NORM group (7.4±4.4 mm, p=0.04, Cohen’s d=0.82). Effusion synovitis significantly correlated with matrix metalloproteinase-3 (rho=0.63, p<0.001), matrix metalloproteinase-1 (rho=0.50, p=0.003), and sulfated glycosaminoglycan (rho=0.42, p=0.01). No other significant correlations were present.

Conclusion

Effusion synovitis was significantly greater for those that demonstrated a dysregulated inflammatory response after acute ACL injury than those with a more normal response to injury. Effusion synovitis was also found to significantly correlate with synovial fluid concentrations of degradative enzymes and a biomarker of early cartilage degradation. Future work is needed to determine if non-invasive methods, such as MRI or ultrasound, may accurately identify patients within this pro-inflammatory phenotype and whether this subset is more prone to more rapid PTOA changes after injury.

## Introduction

While anterior cruciate ligament (ACL) reconstruction restores joint stability, the procedure does not universally protect against the development of posttraumatic osteoarthritis (PTOA). Up to 50% of patients demonstrate symptomatic and irreversible cartilage loss within 10 years after ACL reconstruction [1]. The progression to PTOA after an ACL injury is likely multifactorial, involving biological, mechanical, and psychosocial factors [2]. There is an urgent need to identify those at greatest risk of accelerated progression to PTOA in order to identify novel treatment targets and create evidence-based interventions for this high-risk group.

Following acute joint trauma, there appears to be a subset of patients that demonstrate a dysregulated inflammatory response [3,4]. This pro-inflammatory phenotype, or “Inflamma-type”, is characterized by an amplified pro-inflammatory response combined with a lack of attendant anti-inflammatory response, and has been observed in following both ACL injury and intra-articular fracture [3,4]. This unchecked inflammatory response was independent of time from injury [3] and involves increased synovial fluid concentrations of pro-inflammatory cytokines (interleukin-6 (IL-6) and IL-1β) and degradative enzymes (matrix metalloproteinase-1 (MMP-1) and MMP-3) that likely play active roles in the onset and progression of PTOA [5].

For those with established knee osteoarthritis, effusion synovitis assessed via magnetic resonance imaging (MRI) has been shown to correlate with pain and patient-reported function [6]. Similarly, histological evidence of synovitis after a meniscus injury correlates with both preoperative pain and patient-reported function [7,8]. However, it remains unclear if there is a connection between the dysregulated inflammatory response and synovitis after an ACL injury. The aims of this study were to: 1) compare magnetic resonance imaging (MRI)-measured effusion synovitis between those with vs. without a dysregulated inflammatory response, and 2) assess the correlations between effusion synovitis and synovial fluid concentrations of proinflammatory cytokines, degradative enzymes, and synovial fluid biomarkers of cartilage degradation. We hypothesized that effusion synovitis early after an ACL injury would be greater for the adverse Inflamma-type after an ACL injury and significantly correlate with synovial fluid concentrations of proinflammatory cytokines, MMPs, and biomarkers of cartilage degradation.

## Materials and methods

Study design

This study is a secondary analysis of 35 ACL-injured subjects (15 females, 20 males; age = 19.5 ± 4.4 years, body mass index (BMI) = 24.0 ± 3.2 kg/m^2^) enrolled at one site as part of a multicenter randomized trial (ClinicalTrials.gov ID: NCT01692756) [9]. All patients provided informed consent prior to participating in this IRB-approved trial (University of Kentucky, approval # 45814). From the original study, cluster analysis of synovial fluid samples from 35 patients identified two phenotypic clusters of patients: a pro-inflammatory phenotype (“Inflamma-type”) and a more normal inflammatory response phenotype (NORM group). The current analyses involve baseline data collected prior to any study treatment being administered. As such, a priori power analysis was not performed for this exploratory secondary analysis.

Participants

Skeletally mature patients between the ages of 14 and 32 years with an acute ACL injury that occurred during sports activities were eligible for the study [9]. Exclusion criteria included previous contralateral knee ligamentous or meniscus injury, previous traumatic ipsilateral knee injury and/or surgery, or clinical evidence of posterior cruciate ligament injury or more than grade 1 medial or lateral collateral ligament injury. Patients were also excluded if the injury occurred more than eight days prior to enrollment. Patients were not excluded based on biological sex, race, ethnicity, or the presence of either concomitant meniscus or articular cartilage injury. 

Synovial fluid biomarkers

Synovial fluid was aspirated from the involved knee on the day of enrollment and centrifuged at 3500 RPM for 10 minutes. The supernatant was aliquoted and stored at -80° C until analyzed. Samples were shipped to the Duke University Biomarker Core Facility for analysis at the conclusion of the study [9]. The mean time between injury and joint aspiration was 4.3 days (range = 1 to 8 days). Synovial fluid concentrations of degradative enzymes, cytokines, and biomarkers of cartilage degradation, bone turnover, and hemarthrosis were assessed by investigators blinded to the knowledge of clinical outcomes as previously described [3,9]. The degradative enzymes assessed included MMP-1, MMP-3, and MMP-9 [10,11]. Cytokines evaluated consisted of IL-1α, IL-1β, and IL-1 receptor antagonist (IL-1RA). Synovial fluid biomarkers of cartilage degradation included C-terminal crosslinked telopeptide of type II collagen (CTX-II) [12], cartilage oligomeric matrix protein (COMP) [13], and sulfated glycosaminoglycan (sGAG) [10]. N-terminal crosslinked telopeptide of type I collagen (NTXI) was included as a biomarker of bone remodeling [14], and bilirubin/biliverdin concentration was included as a biomarker of hemarthrosis [10].

MRI measurements of effusion synovitis

**Figure 1:**
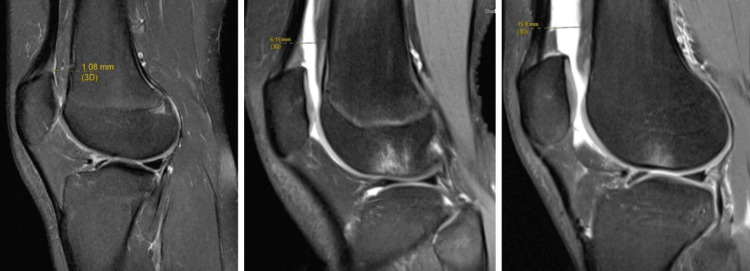
Representative examples of patients with low, moderate, and high-grade effusion-synovitis measurements Low grade - Left; Moderate grade - Middle; High grade - Right

Effusion synovitis was measured on each patient’s preoperative clinical MRI using the Anterior Cruciate Ligament OsteoArthritis Score (ACLOAS) whole joint scoring system [15]. The mean time between injury and MRI was 2.5 days (range = 1 to 8 days). Effusion synovitis, a continuous variable, was defined as the amount of capsular distension (mm) in the suprapatellar recess on a mid-line sagittal fat-suppressed image (Figure 1). Effusion synovitis has been previously graded based on the magnitude of capsular distension (Grade 0 < 2 mm, 1 = > 2 mm and < 5 mm, 2 = > 5 mm and < 10 mm, 3 = > 10 mm). Measurements were performed by a single evaluator (CAJ) experienced with OA-related MRI assessments [16].

Statistical analyses

In the previous work, a cluster analysis was performed with the synovial fluid biomarker concentrations and patients were categorized into two phenotypic subgroups: the Inflamma-type and NORM phenotypes [3]. The Inflamma-type subgroup (29% of the total sample) demonstrated a dysregulated immune response to ACL injury based on the molecular composition of the synovial fluid within eight days of ACL injury; specifically, the Inflamma-type had significantly higher IL-1Ra, MMP-3, and sGAG but lower MMP-9 than the NORM group. In addition, age, sex, BMI, meniscal status, bone bruise volume, and time from injury did not differ between the Inflamma-type and NORM groups [3].

A Grubbs outlier test was performed to detect outliers within the effusion-synovitis measurements. One extremely high value was identified (23.8 mm, p = 0.01) and this patient’s information was excluded from the analyses. The analyses were then performed with the remaining 34 participants. Effusion-synovitis measures were compared between the Inflamma-type and Normal groups using an independent, two-tailed t-test. Cohen’s d effect size was calculated with an effect size between 0.2 and 0.49 considered small, 0.5 to 0.79 considered moderate, and ≥ 0.8 considered large [17]. In addition, demographic factors were compared based on the grade of effusion synovitis (0 = none, 1 = mild, 2 = moderate, 3 = severe) [15]. Because of the small number of patients with Grade 0 or 1 effusion synovitis, descriptive characteristics of those with varying degrees of effusion synovitis have been reported; however, no statistical analyses were performed due to the lack of statistical power. Finally, Spearman’s rho non-parametric correlations were calculated to evaluate the relationship between effusion synovitis and each of the synovial biomarkers included in the study. Non-parametric correlations were used as synovial fluid biomarker concentrations were not normally distributed (as previously described) [3]. The outlier analysis was performed using the “outliers” package within R [18]. Comparative and correlation analyses were performed using SPSS Statistics 28 (IBM Corp., Armonk, NY). For all analyses, an alpha level of p < 0.05 was considered significant.

## Results

**Figure 2:**
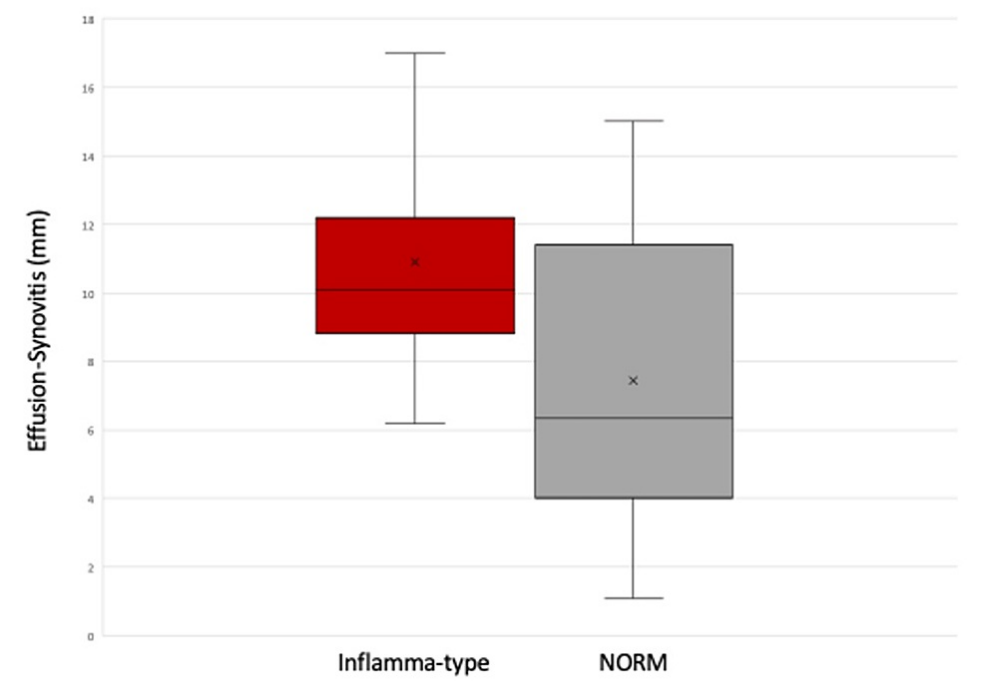
Effusion synovitis (mm) between the pro-inflammatory phenotype (“Inflamma-type”) and those with a more normal inflammatory response phenotype after an anterior cruciate ligament injury (NORM group) For each group, the box represents the interquartile range (IQR), the bar within the box is the median, and the X in the box is the mean. The whiskers represent median ± 1.5 times the IQR.

Most study participants demonstrated moderate (13/34, 38.2%) or severe effusion synovitis (12/34, 35.3%), and demographic and injury characteristics are presented in Table 1. Effusion synovitis on MRI was significantly greater for the Inflamma-type (10.9 ± 3.8 mm) than the NORM-type group (7.4 ± 4.4 mm, p = 0.04, Cohen’s d = 0.82, Figure 2). Effusion synovitis also significantly correlated with individual synovial fluid biomarkers, including MMP-3 (rho = 0.63, p < 0.001), MMP-1 (rho = 0.50, p = 0.003), and sGAG (rho = 0.42, p = 0.01). Consistent with our prior analyses, wherein lower MMP-9 was consistent with the Inflamma-type, MMP-9 was negatively correlated with the effusion-synovitis score, but this result was not significant (p=0.52). No other significant correlations were present (Table 2).

**Table 1:** Demographic and injury characteristics of those with varying degrees of effusion synovitis (Grade 0 = None, 1 = Mild, 2 = Moderate, 3 = Severe) BMI, body mass index

	Synovitis Grade			
Variable	None	Mild	Moderate	Severe
N	2	7	13	12
Female Sex (% F)	1 (50%)	2 (28.6%)	10 (76.9%)	2 (16.7%)
Age	16.5 ± 0.1	20.1 ± 3.2	18.4 ± 3.6	20.7 ± 6.0
BMI	23.6 ± 3.4	23.1 ± 1.8	22.8 ± 2.7	25.4 ± 3.9
Inflamma-type (n (%))	0 (0%)	0 (0%)	6 (46.1%)	4 (33%)
Meniscus injury (n (%))	2 (100%)	6 (85.7%)	12 (92.3%)	11 (91.7%)
Time from Injury (d)	2.5 ± 2.1	2.9 ± 1.8	4.8 ± 2.0	5.0 ± 1.9

**Table 2:** Spearman’s rho correlations between effusion synovitis and synovial fluid biomarkers COMP, cartilage oligomeric matrix protein; CTX-II, C-terminal crosslinked telopeptide of type II collagen; IL, interleukin; MMP, matrix metalloproteinase; NTX-I, N-terminal crosslinked telopeptide of type I collagen; sGAG, sulfated glycosaminoglycan

Synovial Biomarker	Effusion-Synovitis Rho	p
Enzyme Concentrations		
MMP-1	0.50	0.003
MMP-3	0.63	< 0.001
MMP-9	-0.11	0.52
Cytokine Concentrations		
IL-1α	-0.17	0.35
IL-1β	-0.10	0.56
IL-1RA	0.24	0.16
Cartilage Degradation		
COMP	-0.18	0.32
CTX-II	-0.05	0.77
sGAG	0.42	0.01
Bony Remodeling		
NTX-I	19.7 ± 9.8	0.68
Hemarthrosis		
Bilirubin+Biliverdin	0.11	0.53

## Discussion

We previously identified a subgroup with a dysregulated immune response to an ACL injury based on the molecular composition of the synovial fluid within eight days of the ACL injury. Specifically, the Inflamma-type had significantly higher IL-1Ra, MMP-1, MMP-3, and sGAG but lower MMP-9 than the NORM group [3]. In the current analyses, MRI effusion synovitis was significantly greater for those that demonstrate a dysregulated inflammatory response after acute ACL injury than those with a more normal response to injury. Effusion synovitis was also found to significantly correlate with both synovial fluid concentrations of degradative enzymes (MMP-1 and MMP-3) and a biomarker of cartilage degradation (sGAG). Synovitis has been implicated in both osteoarthritis pain and structural progression [6]. In those with established knee osteoarthritis, a phenotype has been identified that is characterized by synovitis and worse pain [19,20]. MRI evidence of effusion synovitis was also shown to be a significant predictor of radiographic knee osteoarthritis both one and two years prior to diagnosis [21].

Increased effusion synovitis for those with a dysregulated inflammatory response after ACL injury may directly influence the initial stages of progressive cartilage changes and subsequent onset of PTOA. Synovitis is a key cog in a vicious cycle leading to early cartilage degradation following joint injury. Injury activates the innate immune response, with macrophages and synoviocytes within the synovium producing pro-inflammatory cytokines [14,22]. This results in the upregulation of MMPs, which then leads to changes in the articular cartilage extracellular matrix (ECM) and the loss of GAG [23,24]. Cartilage breakdown components then further stimulate the innate immune response leading to a cycle of synovitis, inflammation, enzyme activity, and cartilage degradation [22]. While the current study involved a cross-sectional evaluation, the results further support the cycle of inflammation and cartilage degradation as effusion synovitis significantly correlated with both synovial fluid MMP and sGAG concentrations.

**Figure 3:**
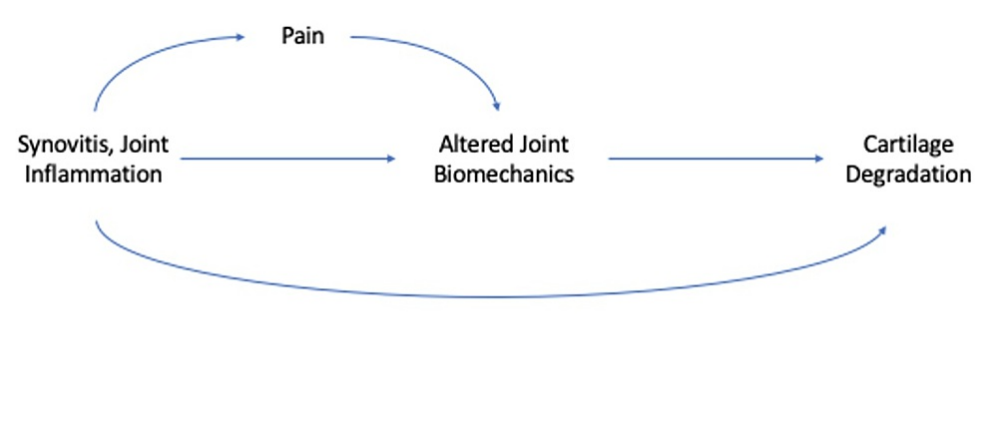
Theoretical time-dependent relationships between synovitis, pain, joint biomechanics, and cartilage degradation after an anterior cruciate ligament injury

A dysregulated inflammatory response may also indirectly influence the progression of PTOA after an ACL injury as part of a multifactorial mechanobiological model. As will be described, there is evidence to suggest that increased synovial fluid cytokine and MMP-3 concentrations are the first step in a chain of events leading to increased pain, altered joint mechanics, and cartilage degradation (Figure 3). Synovitis has been associated with increased pain and increased synovial fluid concentrations of pro-inflammatory cytokines [6]. Those with increased synovial fluid IL-6 and MMP-3 concentrations prior to ACL reconstruction were also demonstrated to have altered joint biomechanics six months after surgery [25]. The relationship between synovial inflammation and joint biomechanics may be further mediated by pain, as pain four weeks after ACL reconstruction was also associated with similar changes in joint mechanics [26]. Altered joint loading six months after ACL reconstruction has then been demonstrated to be associated with progressive cartilage composition changes on MRI during the first three years after ACL reconstruction [27,28].

The relationship between synovitis, synovial inflammatory markers, and PTOA progression may be time-dependent, with synovitis and inflammation perhaps playing more central roles in cartilage degradation during the first two to three years after surgery when compared to later time points. While there is evidence that synovitis and inflammation may increase cartilage degradation early after injury or surgery, this has not been the case at later time points. Neither structural nor molecular biomarkers of inflammation two years after an ACL injury correlated with subsequent radiographic or MRI OA changes at five years [29]. This is consistent with Ingale et al. who reported that different molecular mechanisms were observed at different stages of knee osteoarthritis [30]. The current results fit within this theoretical timeline of interrelated mechanobiological mechanisms of PTOA progression, but well-powered longitudinal mechanistic studies are needed to further evaluate this model.

This study was not without limitations. First, the analyses were performed with 34 patients immediately following an ACL injury. Well-powered longitudinal studies are needed to better understand the interplay between synovitis, the intraarticular joint environment, pain, biomechanics, and cartilage degradation. Second, analyses were performed using standard clinical MRIs. The use of ultrasound and/or contract-enhanced MRI may allow for synovial thickening and effusion to be assessed independently. Third, a limited number of inflammatory markers were evaluated, and proteomic, transcriptomic, metabolomic, and/or other -omic analyses may provide additional insight into the time-dependent mechanisms of PTOA progression after an ACL injury. Finally, the original study employed very narrow inclusion/exclusion criteria, and additional studies will be necessary to determine if these results are generalizable to other age groups or injury patterns.

## Conclusions

Effusion synovitis was significantly greater for those who demonstrated a dysregulated inflammatory response after acute ACL injury than those with a more normal response to injury. Effusion synovitis was also found to significantly correlate positively with synovial fluid concentrations of individual degradative enzymes and is a biomarker of early cartilage degradation. Future work is needed to determine if non-invasive methods, such as MRI or ultrasound, may accurately identify patients within this pro-inflammatory phenotype, and whether this subset is prone to more rapid PTOA changes after injury.
